# Revascularización en diente permanente inmaduro con periodontitis apical usando Neoputty MTA. Reporte de caso

**DOI:** 10.21142/2523-2754-1301-2025-235

**Published:** 2025-03-03

**Authors:** Aleska Irin Cordido Useche, Pedro Rafael Rivero Griman, Mireya Anais Rojas Rocillo

**Affiliations:** 1 Universidad de Carabobo. Valencia, Venezuela. acordido@uc.edu.ve rocillomireya11@gmail.com Universidad de Carabobo Universidad de Carabobo Valencia Venezuela acordido@uc.edu.ve rocillomireya11@gmail.com; 2 Departamento de Estomatoquirurgica, Facultad de Odontologia, Universidad de Carabobo. Valencia, Venezuela. odpedrorivero@gmail.com Universidad de Carabobo Departamento de Estomatoquirurgica Facultad de Odontologia Universidad de Carabobo Valencia Venezuela odpedrorivero@gmail.com

**Keywords:** dentición permanente, periodontitis apical, endodoncia regenerativa, NeoPUTTY MTA, permanent teeth, apical periodontitis, regenerative endodontics, NeoPUTTY MTA

## Abstract

El tratamiento de los dientes permanentes jóvenes necróticos con ápices abiertos representa un reto para los endodoncistas. La revascularización ha surgido como una alternativa de tratamiento con base biológica, que permite el desarrollo continuo de los dientes inmaduros. Este procedimiento amerita un sellado coronal hermético con un cemento biocompatible. El objetivo de este trabajo es exponer un caso de revascularización de un diente inmaduro con ápices abiertos en un paciente de 7 años, en estadio III de Cvek, con el diagnóstico de terapia previamente iniciada y periodontitis apical asintomática, utilizando NeoPUTTY MTA como barrera cervical. Previo consentimiento informado, se siguió el protocolo propuesto por Wei en 2 citas, usando hidróxido de calcio como medicación intraconducto en la primera cita y realizando la punción apical en la segunda cita, previo protocolo de irrigación con activación pasiva ultrasónica. La barrera cervical se creó compactando NeoPUTTY MTA y se cubrió con TheraCal LC más ionómero de vidrio y resina compuesta. El paciente fue evaluado en 4 consultas postratamiento, a las 3 semanas, 6 semanas, 6 meses y 1 año. Se mantuvo asintomático durante todo el proceso y presentó mejoría radiográfica progresiva hasta lograr la resolución de la lesión apical, aumento del grosor de las paredes radiculares, disminución del diámetro apical de la raíz mesial a aproximadamente a 0,5 mm y de la raíz distal aproximadamente a 1 mm, y se formó un nuevo espacio ligamento periodontal.

## Abstract (en)

The treatment of young necrotic permanent teeth with open apices represents a challenge for endodontists. Revascularization has emerged as a biologically based treatment alternative that allows the continued development of immature teeth. This procedure requires a hermetic coronal seal with a biocompatible cement. The objective of this work is to present a case of revascularization of an immature tooth with open apices in a 7-year-old patient, in Cvek stage III, with the diagnosis of previously initiated therapy and asymptomatic apical periodontitis, using NeoPUTTY MTA as a cervical barrier. After informed consent, the protocol proposed by Wei was followed in 2 appointments; using calcium hydroxide as intracanal medication in the first appointment, and performing the apical puncture in the second appointment, after an irrigation protocol with passive ultrasonic activation. The cervical barrier was created by compacting NeoPUTTY MTA and covered with TheraCal LC plus glass ionomer and composite resin. The patient was evaluated in 4 post-treatment consultations, at 3 weeks, 6 weeks, 6 months and 1 year; he remained asymptomatic throughout the process and presented progressive radiographic improvement until resolution of the apical lesion, increased thickness of the root walls, decreased apical diameter of the mesial root to approximately 0.5 mm and of the distal root to approximately 1 mm and the formation of a new periodontal ligament space.

## INTRODUCCIÓN

El objetivo del tratamiento endodóntico no quirúrgico es preservar la dentición natural. Sin embargo, esto se complica cuando individuos jóvenes presentan dientes permanentes inmaduros con necrosis pulpar ^1^. Durante ese periodo, este proceso patológico puede causar la detención del desarrollo radicular, lo que tiene como resultado dientes con paredes débiles y ápices abiertos que dificultan la realización de un tratamiento endodóntico convencional ^2^. La anatomía única de un ápice abierto complica aún más la tarea de obturar completamente el conducto; y el riesgo de que se desborde el exceso de material hacia el periápice plantea desafíos adicionales ^3^^,^^4^.

Las modalidades clásicas de tratamiento para dientes con ápices abiertos incluyen la apexificación mediante la aplicación prolongada de hidróxido de calcio, o la técnica de la creación de una barrera apical. Ambos tratamientos presentan altas tasas de éxito, pero no garantizan la continuidad del desarrollo radicular ^1^. Muchos estudios han evaluado el desempeño de las pastas de hidróxido de calcio y señalan que pueden tener algunas desventajas, como la fragilidad y el alto riesgo de fracturas radiculares ^5^. La apexificación en una sola cita puede lograrse mediante la compactación no quirúrgica de agregado trióxido mineral (MTA) o agentes parecidos, para crear una barrera apical artificial. El MTA fragua en 4-5 horas y está demostrado que promueve la curación del tejido periapical; pero en ápices muy abiertos, puede llegar a extruirse ^6^.

Recientemente, los procedimientos endodónticos regenerativos (PER) han ganado mucha atención como alternativas de tratamiento con una base biológica ^7^. La idea inicial de los PER pertenece a Nygaard Östby, en 1961, quien encontró que el coágulo de sangre tiene el potencial de actuar como relleno del conducto radicular de un diente maduro necrótico en donde el ápice se aumentó de tamaño de manera intencional. Durante los últimos 20 años, los PER se han realizado principalmente como procedimientos terapéuticos para dientes inmaduros con necrosis pulpar y periodontitis apical. Varios estudios han demostrado que pueden conducir a la resolución de los signos y síntomas, el engrosamiento de las paredes radiculares y la continuación de la maduración radicular ^8^. En 2004, Banch y Trope reportaron un caso con un protocolo de revascularización modificado para el cual se creó un coágulo en el conducto después de su desinfección y se colocó una barrera coronal hermética, lo que deja evidencia clínica de la aplicación una endodoncia regenerativa ^9^. Estos procedimientos apuntan a restaurar funciones como la mineralización, la inmunidad y la sensibilidad pulpar. Esta técnica incorpora de manera equilibrada 3 componentes principales: células (principalmente, células madre), moléculas bioactivas (factores de crecimiento) y andamios ^10^. 

Los PER están indicados en casos en los cuales se cumplen los siguientes criterios: a) Diente necrótico permanente con desarrollo radicular incompleto; b) Casos en los que no se necesite un poste para la restauración; c) Pacientes/padres colaboradores y obedientes; d) Ausencia de alergias a los medicamentos. El paciente debe tener un sistema inmunológico competente para el buen control de las infecciones (9). A partir de la clasificación de desarrollo radicular de Cvek, se han propuesto las siguientes recomendaciones: los dientes permanentes con necrosis pulpar en estadio 1 (menos de la mitad de desarrollo radicular y ápice abierto), estadio 2 (desarrollo radicular a la mitad y ápice abierto) y estadio 3 (2/3 de desarrollo radicular y ápice abierto), de preferencia, deben recibir un PER debido a su raíz corta, paredes delgadas y ápices abiertos. Los dientes inmaduros en estadio 4 (desarrollo radicular casi completo y ápice abierto) pueden ser tratados mediante un PER o con una barrera apical de MTA ^11^.

Este tratamiento inicia con la desinfección química de los conductos. A la irrigación le sigue medicación intraconducto con pasta triple antibiótica (PTA) o hidróxido de calcio. Posteriormente, se induce una hemorragia en el conducto radicular para formar un coágulo sanguíneo que aporta factores de crecimiento a las células y actúa como andamiaje, para luego sellar el orificio del conducto con un cemento hidráulico que permite la regeneración de nuevo tejido adyacente al mismo. Finalmente, se coloca la restauración coronal definitiva ^12^. 

El MTA es el material más comúnmente usado como barrera cervical en los PER, según reporta la literatura ^13^. Posee una excelente biocompatibilidad y capacidad de sellado; sin embargo, su extenso tiempo de fraguado, su alta solubilidad en su periodo inicial, la pigmentación dental y su baja fuerza compresiva pueden ser inconvenientes para su manejo, y pueden conducir a fallas en el tratamiento ^14^. En los últimos años se han desarrollado muchos cementos hidráulicos que superan los inconvenientes del MTA, pero conservando propiedades favorables como la actividad antibacteriana, la baja toxicidad y su leve respuesta inflamatoria ^15^. Entre ellos destaca el NeoPUTTY MTA, un material biocerámico, bioactivo, premezclado que ofrece propiedades excepcionales tanto para su manipulación como para la estimulación de la formación de hidroxiapatita. Su consistencia, baja solubilidad y biocompatibilidad minimizan el desperdicio, los costos y el tiempo de trabajo. Esta conjugación de características superiores hace al NeoPUTTY MTA una solución eficiente y el material de elección para los PER ^16^.

Se deben programar citas periódicas para evaluación clínica y radiográfica. Al examen clínico, se debe evaluar la presencia de dolor a la palpación/percusión, inflamación de tejidos blandos y tracto sinuoso. Radiográficamente, se debe observar el estado de la radiolucidez apical y el aumento del grosor y longitud de las paredes radiculares ^17^. Los criterios de éxito comprenden ausencia de dolor, ausencia de signos y síntomas de inflamación, curación de la lesión periapical preexistente, incremento de la longitud y grosor de las paredes radiculares, ausencia de resorción radicular externa, respuesta positiva a las pruebas de sensibilidad, aceptación del paciente, ausencia de decoloración y detección radiográfica de un nuevo espacio del ligamento periodontal a lo largo de la pared interna del conducto radicular ^18^. 

El objetivo de este artículo es exponer un caso sobre una revascularización en un diente inmaduro con ápices abiertos, con el diagnóstico de la Asociación Americana de Endodoncia (AAE) de terapia previamente iniciada con periodontitis apical asintomática usando NeoPUTTY MTA como barrera cervical.

## REPORTE DE CASO

### Consideraciones éticas

La presente investigación fue aprobada por la representante legal del paciente, por ser este menor de edad ante la legislación venezolana, a través de la firma del consentimiento informado que pertenece a la historia clínica del posgrado de Endodoncia de la Facultad Odontología de la Universidad de Carabobo. Dicho documento contempla lo establecido en la Ley de los Derechos Civiles, tercer capítulo, que describe el principio de autonomía de las personas, y en el Código de Deontología Odontológica, artículo 57, al cual añade que toda persona debe manifestar con libertad su voluntad de aceptar o rechazar en su condición de ser paciente, así como también rehusar determinadas indicaciones diagnosticas o terapéuticas.

El caso se presenta siguiendo las recomendaciones del Reglamento de la Comisión de Bioética y Bioseguridad de la Universidad de Carabobo. Adicionalmente, se firmó un consentimiento informado por parte de los padres del paciente.

### Descripción del caso

**Figura 1:**
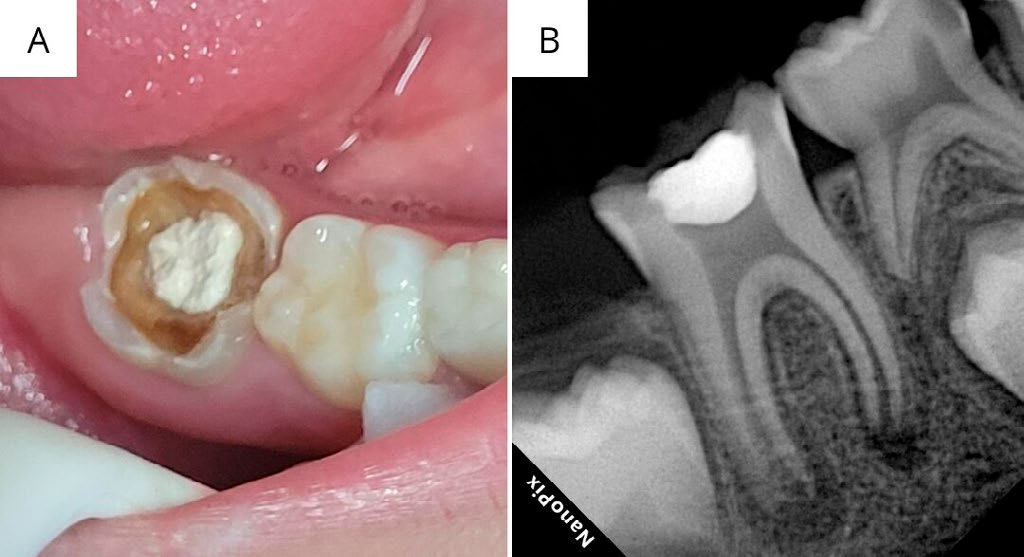
a) Fotografía inicial intrabucal. b) Radiografía inicial.

Masculino de 7 años acude al área de endodoncia para la evaluación de la pieza 46. Sin antecedentes médicos relevantes ni medicación actual. Aparentemente, de comportamiento colaborador. A la exploración clínica intrabucal, no refiere dolor a la percusión lateral, vertical ni a la palpación periapical. La cámara está cerrada con un cemento provisional de óxido de zinc con eugenol (20E CURE®, Secure, Venezuela) (Figura 1A). Las pruebas de sensibilidad al frío y calor fueron negativas, con sondaje normal. Mediante la exploración radiográfica, se observó a nivel coronal una imagen radiopaca compatible con restauración (Figura 1B). A nivel apical, se observó un ensanchamiento del espacio del ligamento periodontal, con radiolucidez de 4,2 x 4,5 mm (distal) y 3,8 x 3,5 mm (mesial). El diámetro apical radiográfico es, aproximadamente, de 1 mm en la raíz mesial y de 2 mm en la raíz distal.

Se diagnosticó la pieza 46 con terapia previamente iniciada y periodontitis apical asintomática, según criterios de la AAE. De acuerdo con las características del paciente, diente permanente inmaduro sin vitalidad pulpar con ápice abierto en estadio III de Cvek ^11^. Según el algoritmo de tratamiento propuesto por Murray ^19^, se decidió realizar un PER de tipo revascularización. 

**Figura 2:**
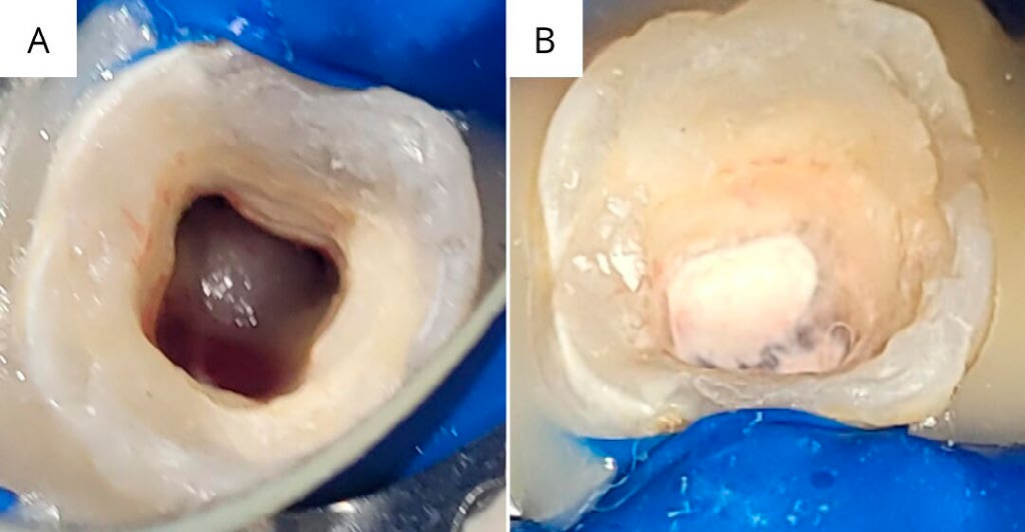
a) Hemorragia intraconducto inducida. b) Barrera apical de NeoPUTTY MTA.

Se siguió el protocolo propuesto por Wei ^9^ y se infiltró lidocaína al 2%. Se llevó a cabo la remoción de provisional con fresa redonda, bajo aislamiento absoluto. La neutralización fue hecha con NaOCl al 3,25%. Se realizó una conductometría, seguida por un ligero raspado de las paredes con lima manual 20,02. Se colocó hidróxido de calcio en polvo más glicerina como medicación intraconducto y se selló de manera provisional con ionómero de vidrio (i-LINER® Light Curing Compomer Liner, i-Dental, Lituania). En la segunda cita, se anestesió con lidocaína al 2%, se removió el provisional y la medicación intraconducto, bajo aislamiento absoluto El protocolo de irrigación se inició con la activación pasiva ultrasónica usando NaOCl al 3,25% (3 ciclos de 20 segundos), solución fisiológica al 0,9%, 20 cc EDTA al 17% x 5 min, 5 cc de NaOCl al 3,25% y solución fisiológica al 0,9%. La punción de la papila apical se realizó con una lima 25,02 y se logró la inducción al sangrado (figura 2A). Se procedió a la creación de barrera cervical compactando NeoPUTTY MTA (NeoPUTTY® By Avalon Biomed, ZARC, TX, EE. UU.) (Figura 2B). Se cubrió con TheraCal LC (TheraCal LC®, BISCO, EE. UU.), más ionómero de vidrio y resina compuesta. 

**Figura 3:**
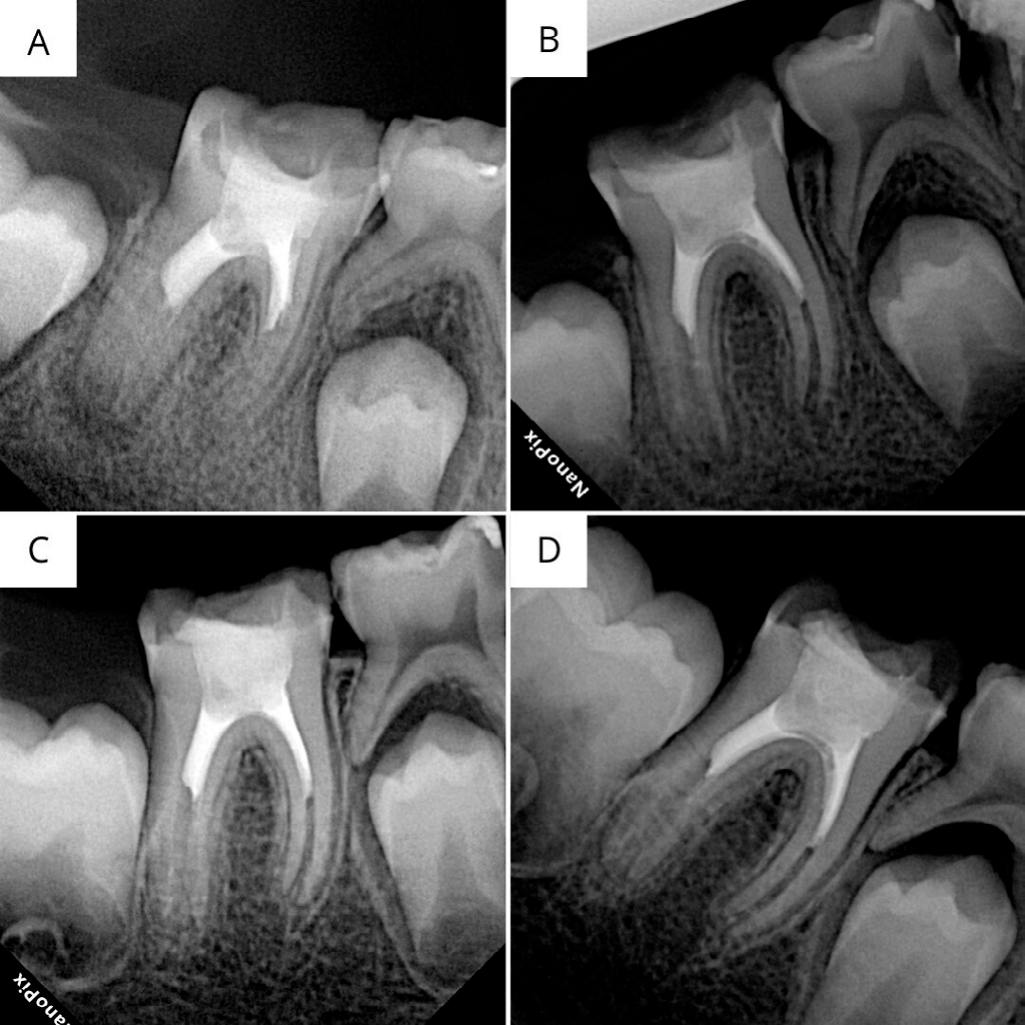
a) Control radiográfico a las 3 semanas. b) Control radiográfico a las 6 semanas. c) Control radiográfico a los 6 meses. d) Control radiográfico a los 12 meses.

El paciente fue evaluado en 4 consultas postratamiento, a las 3 semanas, 6 semanas, 6 y 12 meses, en las cuales se evaluaron síntomas y signos clínicos y radiográficos, según los criterios de la Sociedad Europea de Endodoncia ^18^. En cuanto a síntomas y signos clínicos, el paciente se mantuvo asintomático durante todo el proceso. Radiográficamente, a las 3 semanas se observó resolución de la lesión periapical en la porción mesial y disminución de 2 mm de la lesión ósea preexistente en la porción distal; sin cambios radiculares ni evidencia radiográfica de un nuevo ligamento periodontal (Figura 3A). En el control de las 6 semanas se observó la resolución de la lesión periapical en la porción mesial y una disminución de 4 mm de la lesión ósea preexistente en la porción distal, con aumento del grosor de las paredes radiculares (Figura 3B). Se le indicó que llamara si presentaba algún tipo de dolor o desarrollaba algún tracto sinuoso. En el control de los 6 meses, se observó resolución de la lesión periapical ósea preexistente, aumento del grosor de las paredes de las raíces, disminución del diámetro apical de la raíz mesial a aproximadamente 0,5 mm y de la raíz distal a aproximadamente 1 mm. Asimismo, se detectó un nuevo espacio ligamento periodontal a lo largo de la raíz mesial (Figura 3C). La prueba de sensibilidad al frío dio positiva en la cita de los 6 meses. En el control a los 12 meses, se observó resolución de la lesión periapical ósea y el hallazgo más resaltante fue la detección de un nuevo espacio ligamento periodontal a lo largo de la raíz distal (Figura 3D). La prueba de sensibilidad al frío dio positiva en la cita de los 12 meses.

## DISCUSIÓN

El tratamiento endodóntico de dientes necróticos inmaduros con formación radicular incompleta es extremadamente complicado. Los PER han sido reportados en muchos estudios clínicos como un nuevo tratamiento con buena eficacia en la promoción del desarrollo radicular continuo ^6^. La morfología de la raíz también debe considerarse en la selección de los casos. Estudios recientes demostraron que el mayor incremento en el engrosamiento radicular, la longitud y el estrechamiento apical fue encontrado en los dientes con diámetros apicales preoperatorios ≥ 1 mm ^9^. En el caso reportado, el diámetro apical preoperatorio era mayor a 1 mm y se observó un aumento considerable del grosor de las paredes, así como una disminución del diámetro apical de la raíz mesial a 0,5 mm y de la raíz distal a 1 mm, aproximadamente.

El éxito del tratamiento de revascularización depende de tres aspectos: la desinfección, la presencia de un andamio (coágulo de sangre) y un sellado coronario hermético ^3^. Se han utilizado diversos medicamentos para la desinfección de los conductos radiculares en los PER, entre ellos la pasta triple antibiótica (TAP) con diferentes combinaciones, la pasta doble antibiótica (DAP) y el Ca(OH)2. El Ca(OH)2 tiene varias ventajas sobre la TAP, entre ellas la ausencia de decoloración, una menor citotoxicidad para las células madre, una mayor supervivencia y proliferación de células madre en la dentina tratada, la promoción de la liberación de factores de crecimiento de la dentina tratada y una eliminación más fácil de los conductos radiculares; por lo cual, la ESE ha recomendado el hidróxido de calcio como medicamento intraconducto para los PER.

Trevino *et al*. investigaron los posibles efectos nocivos que pueden tener los diferentes irrigantes sobre las células pluripotenciales y concluyeron el EDTA al 17% promueve la supervivencia de estas, por lo que este protocolo resultó muy beneficioso para los procedimientos de revascularización ^20^. En el presente estudio, se realizó el protocolo de irrigación propuesto por Wei ^9^ usando EDTA al 17% y se obtuvo resultados satisfactorios. La proliferación y diferenciación de células mesenquimales en el conducto radicular es la responsable de la formación, la reparación y la regeneración tisular. Por consiguiente, está indicado que el irrigante permanezca en contacto con las células mesenquimales durante un período de tiempo adecuado para promover la acción antimicrobiana, sin interferir en la diferenciación y proliferación de estas células. Se ha demostrado que la tasa de supervivencia de las células mesenquimales es mayor cuando se emplea el hipoclorito de sodio en bajas concentraciones, lo que aumenta la posibilidad de un proceso de revascularización exitoso ^21^. Durante el protocolo de irrigación final utilizado en el caso reportado, se utilizó NaOCl al 3,25% en orden de preservar la viabilidad de las células pluripotenciales ^3^. En los casos de PER, se recomienda una instrumentación mínima para eliminar la biopelícula, sin debilitar las paredes del conducto radicular ^11^. En nuestro caso, se realizó un ligero raspado de las paredes con una lima K 20,02. 

Las guías recomiendan para el sellado hermético de la porción coronal el uso de un material como el MTA, que debe aplicarse de 2 a 3 mm por debajo de la unión cemento esmalte ^15^. El MTA es un material comúnmente utilizado en procedimientos de revascularización, pero presenta ciertos inconvenientes ^3^. Bartaw *et al*. mencionan que las principales desventajas del MTA incluyen la dificultad para su manipulación y la posibilidad de decoloración del diente, particularmente cuando se aplica en la región coronal ^22^. El MTA tiene un tiempo de fraguado más prolongado (2 horas 45 minutos) en comparación con otros materiales (Biodentine, 12min; TotalFill, 2h; PCM, 3min). Por lo tanto, el MTA permanece poroso por más tiempo incrementando su absorción de sangre, con su consecuente decoloración ^23^. El NeoPUTTY MTA es un material biocerámico premezclado, listo para usar, que fue diseñado para superar casi todas las desventajas presentes en el MTA tradicional. Al ser un material relativamente nuevo en odontología, existen pocos estudios disponibles sobre su uso. Sin embargo, se ha demostrado que posee propiedades mecánicas y físicas similares o incluso superiores a otros materiales, incluyendo una mayor resistencia a la compresión, un mejor tiempo de fraguado y una menor solubilidad ^24^. Entre sus ventajas se encuentra su fácil manipulación, lo que reduce el tiempo en el consultorio y minimiza el riesgo de errores de procedimiento. También exhibe excelentes propiedades de sellado y biocompatibilidad, lo que promueve la curación y regeneración exitosa de los tejidos. Su rápido tiempo de fraguado agiliza los procedimientos y lo hace particularmente beneficioso en emergencias, o cuando se requiere una intervención rápida. En general, las propiedades de su uso sencillo junto con sus capacidades de sellado y biocompatibilidad lo convierten en un material práctico y eficaz para su uso en diversos procedimientos endodónticos ^3^. Todas estas propiedades descritas anteriormente lo convirtieron al NeoPUTTY MTA en el material de sellado coronal de elección para la realización del procedimiento de revascularización reportado en este caso.

Para considerar exitoso un PER, la AAE describe las siguientes metas clínicas: en primer lugar, la eliminación de síntomas y signos clínicos y la evidencia de reparación de la lesión ósea; en segundo lugar, el desarrollo radicular continuo; y, finalmente, el restablecimiento de la neurogénesis o respuesta positiva a las pruebas de sensibilidad ^11^. Los resultados obtenidos en el caso reportado, en un periodo de evaluación postratamiento de al menos 12 meses de seguimiento, están en línea con estos objetivos descritos en la literatura, con lo que se logran de manera satisfactoria todas las metas.

## CONCLUSIÓN

La revascularización representa una alternativa prometedora para el tratamiento de los dientes permanentes inmaduros con ápices abiertos, ya que permite la continuación del desarrollo radicular y el mantenimiento de la vitalidad pulpar. Una de las etapas primordiales para el éxito de un PER es el sellado coronal hermético con un cemento biocompatible. El presente caso muestra resultados satisfactorios, en un seguimiento de 12 meses, en la resolución de las lesiones preexistentes y el mantenimiento de la vitalidad y continuidad de desarrollo radicular de un diente usando NeoPUTTY MTA con material de sellado coronal. Se observan características favorables de este cemento, como su tiempo de fraguado corto, fácil manipulación, excelente biocompatibilidad y capacidad de sellado sin riesgo de decoloración dental, lo cual lo coloca como una alternativa para la realización de estos procedimientos.

## Financiamiento:

Ninguno
